# Bilateral extramidline involvement in intracranial germinoma: diagnostic lessons from a complex presentation

**DOI:** 10.1055/s-0046-1816037

**Published:** 2026-02-27

**Authors:** Fei He, Renato P. Munhoz, Suneil K. Kalia, Leah M. Skory, David F. Tang-Wai

**Affiliations:** 1 University of Toronto, Temerty Faculty of Medicine, Department of Medicine, Division of Neurology, Toronto ON, Canada.; 2 University Health Network, Toronto Western Hospital, Krembil Brain Institute, Toronto ON, Canada.; 3 University of Toronto, Temerty Faculty of Medicine, Department of Surgery, Division of Neurosurgery, Toronto ON, Canada.; 4 Barrie Community Health Centre, Barrie ON, Canada.

**Keywords:** Germinoma, Medical Oncology, Homeopathic Semiology

## Abstract

A 25-year-old man presented with subacute progressive anxiety, cognitive deficits, hypernatremia, and left hemiparesis. Magnetic resonance imaging demonstrated multifocal lesions in bilateral basal ganglia (globus pallidus and putamen), fornices, pineal region, along with Wallerian degeneration of the right corticospinal tract with right brainstem atrophy. Serum B-hCG, alpha-fetoprotein, alkaline phosphatase, and placental alkaline phosphatase were normal. Cerebrospinal fluid studies were unremarkable. Brain biopsy revealed intracranial germinoma. The case herein presented highlights a rare presentation of an uncommon neuro-oncological condition that often demonstrates excellent treatment response but is often diagnosed late due to its non-specific presenting symptoms.

## CLINICAL VIGNETTE

A 25-year-old Caucasian man with a medical history of migraine and childhood asthma presented with a 9-month history of subacute progressive symptoms. Initially, he experienced anxiety, which he attempted to self-medicate with cannabis. Over the following 3 months, he developed cognitive difficulties, including impaired organization of daily tasks such as arranging his clothes and packing his lunch. During this time, routine bloodwork revealed new, persistent hypernatremia, ranging from 146 to 154 mmol/L (reference range [RR] 135–145 mmol/L). He was evaluated by nephrology and diagnosed with central diabetes insipidus, for which desmopressin was initiated. Three months later, his family reported anterograde memory impairment, characterized by repetitive speech and repeated tasks, such as feeding his dog multiple times per day. By the 9th month, he was struggling to perform at work, started to have headaches with nausea despite discontinuing cannabis, and developed subacute left hemiparesis and mild left hemibody sensation deficit, prompting referral to and admission under neurology.

On neurological examination, the patient exhibited apathy and difficulties with delayed recall. He scored 23/30 on the Montreal Cognitive Assessment, with impairments in both frontal executive and anterograde memory functions. Cranial nerve exam was normal with no visual field deficits and no papilledema on dilated fundoscopic examination. Motor examination revealed left arm and leg spasticity, pyramidal pattern of weakness, sustained ankle clonus, brisk reflexes with cross adductor response, left Hoffman and bilateral Babinski signs.

**Figure 1:**
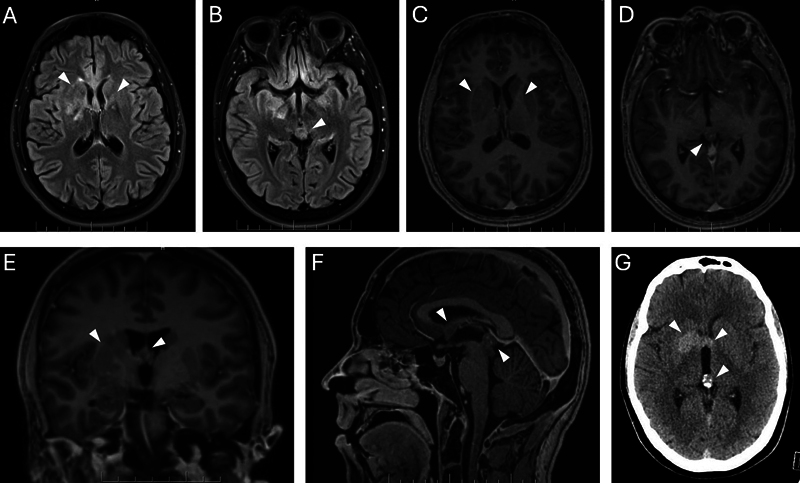
Magnetic resonance imaging (MRI) of intracranial germinomas in midline suprasellar and pineal regions, bilateral basal ganglia, fornices and hippocampi. Axial T2-fluid-attenuated inversion recovery-weighted MRI demonstrating areas of ill-defined T2-hyperintense lesions involving bilateral (R > L) basal ganglia, fornices, and pineal gland ( **A,B** ). Axial and sagittal post-contrast T1 imaging ( **C–F** ) demonstrating mass lesions in bilateral fornices, hippocampi, suprasellar region, and pineal gland, with mild heterogeneous enhancement basal ganglia and pineal gland. Azial brain computed tomography ( **G** ) showing corresponding hyperdensity, as well as calcifications seen in the pineal gland.

**Figure 2:**
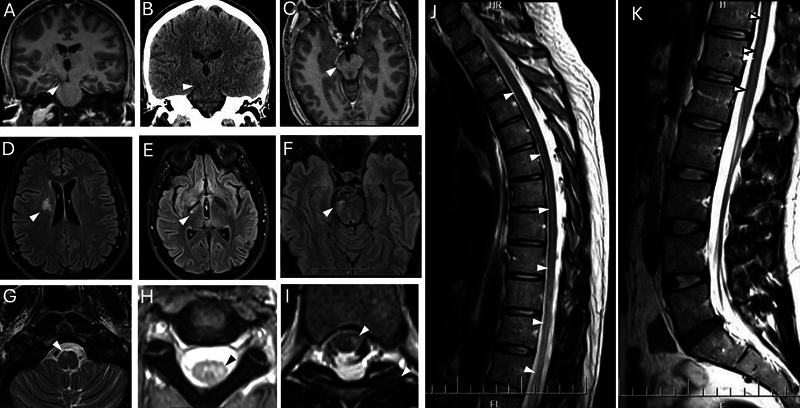
Extensive Wallerian degeneration of corticospinal tract secondary to intracranial germinoma. Coronal post-contrast T1 magnetic resonance imaging (MRI) brain exhibiting hypointensity along the right corticospinal tract from internal capsule to brainstem ( **A** ) with corresponding hypoattenuation on brain computed tomography ( **B** ), and associated hemiatrophy of right midbrain ( **C** ). Axial brain and spine MRI demonstrating T2-hyperintensity along the entire right corticospinal tract extending from right centrum semiovale ( **D** ), right cerebral peduncle ( **E** ), right pons ( **F** ), right medullary pyramid ( **G** ), where it decussates and continues to extend down to the left cervical and thoracic cord ( **H,I** ). Sagittal T2-weighted MRI of spine showing T2-signal changes along the corticospinal tract in lower cervical, thoracic and lumbar spine ( **J,K** ).

Magnetic resonance imaging of the brain and spine demonstrated a T2-hyperintense lesion in the pineal gland and multi-focal faintly heterogeneously enhancing T2-signal hyperintense lesions in the bilateral right more than left basal ganglia (predominantly globus pallidus and putamen), fornices, and hypothalami ( Figure 1A–F ). These areas correspond to hyper-attenuation on computed tomography (CT) brain ( Figure 1G ) indicating a tumor rather than an inflammatory process. In addition, there was continuous non-enhancing T2/fluid-attenuated inversion recovery (FLAIR) hyperintensity extending from the right frontal centrum semiovale to the basal ganglia, through the right corticospinal tract to pons, medulla, decussating into the contralateral cervical spinal cord and down the entire spinal cord ( Figure 2 ). This later radiological sign extension through the thoracic and lumbar spinal cord likely represents Wallerian degeneration of the corticospinal tracts secondary to extensive intracranial germinoma involvement, rather than direct tumor infiltration. This interpretation is supported by associated hypoattenuation on CT and hemiatrophy of the right brainstem ( Figure 2 ). Visual field testing using the 24-2 Humphrey protocol was within normal limits in both eyes. Optical coherence tomography assessment of the retinal nerve fiber layer and ganglion cell complex also demonstrated normal thickness bilaterally.

The patient's serological assessment was normal and included complete blood count, liver, renal, thyroid function tests, lactate dehydrogenase, anti-neutrophil cytoplasmic antibodies (ANCA), erythrocyte sedimentation rate (ESR), c-reactive protein (CRP), rheumatoid factor, and complement levels. Additional testing, including serum protein electrophoresis, syphilis and hepatitis panels, morning cortisol and testosterone levels, IgG4, AQP4, and MOG antibodies, and autoimmune and paraneoplastic panels (serum and CSF), was normal or negative. His serum β-hCG was < 1 IU/L (RR < 3), alpha-fetoprotein 6.6 ug/L (RR ≤ 8.9), alkaline phosphatase 49 U/L (RR 40–150), and placental alkaline phosphatase < 20 U/L (RR 38–126).

Spinal fluid examination revealed an opening pressure of 15 cm H2O, normal protein, cell count and differential with negative culture, viral, and fungal cultures. Oligoclonal bands were positive at 15 (RR < 2) with elevated IgG index of 87 (< 70). Cytology and flow cytometry were negative. CSF β-hCG was 3.55 IU/L, alpha-fetoprotein was 2.7 ug/L, alkaline phosphatase < 10 U/L, and lactate dehydrogenase < 30 U/L. Comprehensive autoimmune and paraneoplastic panels in serum and CSF were negative (including AMPRAR1/R2, CASPR2, DPPX, GABA-R B1,2, LGI1, NMDAR, amphyphysin, CV2, GAD65, Hu, Ma2:Ta, SOX1, Recoverin, Ri, Titin, DNER, Yo, Zic4, and GAD54). He underwent a stereotactic needle biopsy of the right putamen and globus pallidus lesion with pathology consistent with an intracranial germinoma. Namely, microscopic assessment of the specimen showed a malignant neoplasm forming sheets with an intense lymphohistiocytic inflammatory infiltrate in the background. The tumor cells showed round to polygonal nuclei, prominent nucleoli, and eosinophilic clear cytoplasm with mitotic figures. Immunohistochemistry demonstrated weak, focal positive AE1/AE3, positive SALL4, OCT3/4 and CD117, with negative glypican 3, B-HCG, and CD30. Following the diagnosis, the patient was started on 4 cycles of chemotherapy with carboplatin and etoposide over 21 days, followed by radiation therapy with intent to cure.

Since completing therapy, the patient has experienced gradual improvements in energy, allowing greater participation in daily activities such as grocery shopping. Anxiety has slightly improved, particularly on nights of restorative sleep. He has gained meaningful psychological insight, recognizing that his tumor-related deficits contributed to his prior job termination, which he now describes as a “freeing” realization. Neurologically, he demonstrates incomplete but significant improved strength and sensation in the left hemibody. He ambulates independently without aids, compensating for left foot drop with hip hiking, and maintains functional use of the left upper extremity despite slower coordination, which he actively trains through music practice. Cognitive impairments, especially with short-term memory and new learning, remain significant. Surveillance imaging shows no evidence of residual or recurrent intracranial or spinal disease, with only expected post-treatment changes noted.

## FROM PRESENTATION TO RESOLUTION: LESSONS LEARNED

### How can early, non-specific symptoms be anatomically localized in intracranial germinomas?

Intracranial germinomas are slow growing tumors that most commonly occur in children and adolescents. It reaches peak diagnosis in the 2nd decade of life, with a male predominance, and accounts for 0.3 to 3.4% of all brain tumors and 50% of all central nervous system (CNS) germ cell tumours. 1 They often present initially with non-specific symptoms including headache, nausea, neuropsychiatric and cognitive disturbance, and endocrinopathy. Clinical findings often correlate closely with tumor location and size; however, the time from initial symptom onset to diagnosis remains variable from 1 month to 4.5 years with average period of 1.5 years. 2 3 Although germinomas have excellent response to chemo- and radiation therapy with a 5-year overall survival rate of 98.6% and progression-free survival of 87.3%, 4 early diagnosis reduces mortality and significantly reduces morbidity in young patients by reducing extent of tumor infiltration and level of radiation therapy and its related long-term complications. 2

### What are the typical and atypical anatomical sites of intracranial germinomas?

In general, germinomas are considered to originate from primordial germ cells entrapped during stages of embryogenic migration. As a result, they typically occur in midline supratentorial structures in the pineal gland (50%) and suprasellar region (20–30%). 5 Less commonly, germinomas also occur in the basal ganglia and thalamus (6–10%), most unilaterally. 5 Isolated germinoma in fornix and mammillary body have also been reported. 6 Germinomas affecting the basal and thalamus are often associated with ipsilateral cerebral and brainstem hemiatrophy, which is due to tumor infiltration into the thalamocortical and corticospinal tract with subsequent induction of anterograde (Wallerian) and retrograde degeneration. 7 8 9

### How can germinomas mimic inflammatory or autoimmune disorders?

The patient described here had CSF findings—particularly the presence of oligoclonal bands—that can resemble those seen in autoimmune or inflammatory disorders contributing to diagnostic complexity. However, germinomas have been reported in association with oligoclonal bands, further complicating differentiation from immune-mediated conditions. 10 11 12 In this case, however, the imaging features were not consistent with a primary autoimmune or inflammatory disorder, with the possible exception of sarcoidosis.

### What makes this case unique in the spectrum of germinomas?

The case herein discussed describes the first documentation of intracranial germinoma that synchronously involve the bilateral fornix, basal ganglia, and midline suprasellar and pineal regions. It also illustrates a rare extensive involvement of the corticospinal tract extending from the frontal centrum semiovale down the spinal cord. Clinically, the patient initially presented with 3 months of anxiety prior to the onset of frontal-dysexecutive syndrome, with the initial symptom being a red flag that can sometimes be overlooked. This sequence of presentation is due to progressive involvement of the basal ganglia, followed by diabetes insipidus from hypothalamic involvement, memory loss from forniceal and Papez circuit involvement, and eventual hemiparesis from involvement of the posterior internal capsule with subsequent Wallerian degeneration of the corticospinal tract.

### What are the key diagnostic lessons from this case?

The diagnostic challenges in this case were attributable less to disease-specific early indicators and more to limitations in interpretation and anatomical localization. In retrospect, two key considerations emerge:

- closer attention to precise anatomical localization on the initial MRI, and
- systematic correlation of clinical signs with the observed imaging abnormalities.

While neither factor would have specifically indicated a germinoma, both could have supported earlier recognition of an underlying pathological process and prompted more timely diagnostic evaluation.

The patient's presentation, though seemingly fragmented across the psychiatric, cognitive, autonomic, and motor domains, reflects a unifying lesion at the core. Just as distinct clinical signs can appear disparate, this case highlights that they may, in fact, stem from a unified underlying mechanism.

## Notes

TEACHING POINTS

1. Intracranial germinomas, which often occur in adolescents and young adults, may present with non-specific symptoms such as mood changes, anxiety, irritability, headache, and nausea.
2. Intracranial germinomas typically arise in midline supratentorial structures, such as the pineal gland and suprasellar region, but may also occur off-midline in areas like the basal ganglia, thalamus, and fornix.
3. Wallerian degeneration of the corticospinal tract with brainstem hemiatrophy, extending from the centrum semiovale, may accompany germinomas involving the basal ganglia, thalamus, and the pineal or pituitary regions.
